# Comprehensive, Population-Based Sensitivity Analysis of a Two-Mass Vocal Fold Model

**DOI:** 10.1371/journal.pone.0148309

**Published:** 2016-02-04

**Authors:** Daniel Robertson, Matías Zañartu, Douglas Cook

**Affiliations:** 1 Division of Engineering, New York University–Abu Dhabi, Abu Dhabi, United Arab Emirates; 2 Department of Electronic Engineering, Universidad Técnica Federico Santa María, Valparaiso, Chile; The University of Chicago, UNITED STATES

## Abstract

Previous vocal fold modeling studies have generally focused on generating detailed data regarding a narrow subset of possible model configurations. These studies can be interpreted to be the investigation of a single subject under one or more vocal conditions. In this study, a broad population-based sensitivity analysis is employed to examine the behavior of a virtual population of subjects and to identify trends between virtual individuals as opposed to investigating a single subject or model instance. Four different sensitivity analysis techniques were used in accomplishing this task. Influential relationships between model input parameters and model outputs were identified, and an exploration of the model’s parameter space was conducted. Results indicate that the behavior of the selected two-mass model is largely dominated by complex interactions, and that few input-output pairs have a consistent effect on the model. Results from the analysis can be used to increase the efficiency of optimization routines of reduced-order models used to investigate voice abnormalities. Results also demonstrate the types of challenges and difficulties to be expected when applying sensitivity analyses to more complex vocal fold models. Such challenges are discussed and recommendations are made for future studies.

## Introduction

The mechanism by which human voice is produced is incredibly complex, consisting of multiple material and geometric nonlinearities [1, 2]. Furthermore, the system which produces voice (vocal folds, glottis, trachea, etc.) is very difficult to access for the purpose of data collection, thus making the acquisition of experimental data very difficult. These challenges have led many researchers to use numerical models to investigate certain aspects of human phonation. In general, the purpose of these models is to provide insights regarding the actual physical system which they represent. However, the behavior of even the simplest vocal fold models involves complex, nonlinear interactions and multidimensional solution spaces containing numerous discontinuities and bifurcations [3–6]. These complexities can obscure important relationships and present an obstacle to obtaining a comprehensive understanding of overall model behavior(s) that can be generalized to the human population. In fact, the global behavior of the vast majority of vocal fold models has never been fully characterized.

Numerical vocal fold studies have generally focused on generating detailed data regarding a narrow subset of model configurations. For example, a number of studies have examined lumped mass vocal fold models using bifurcation diagrams and regime plots to study model effects across two parameters [1, 7]. This approach serves to illustrate regions with similar kinematic behavior. However, it fails to capture interactions with other parameters, which (as shown later) are highly influential in this system. A partial sensitivity analysis of a two-mass model was performed by Sciamarella and d’Alessandro [8], in which they analyzed 7 out of 21 input parameters using pairwise variations. The role of model parameters in certain topics of interest, such as the effect of acoustic coupling, the ability to reproduce common ranges of phonation, and the prevalence of bifurcations, was investigated. However, an *a priori* ranking of parameter sensitivities was used to neglect the influence of two-thirds of the parameter set, multi-parametric interactions were neglected, and a methodology for performing a more comprehensive sensitivity analysis was not discussed. Using a finite element model, Pickup and Thomson [9] used a screening method to explore the role of 13 geometric factors of a finite element model of vocal fold vibration. However, all 7 material parameters were held constant, and the screening method used by Pickup and Thomson utilized a small number of simulations (28), with the goal of having a first-order approximation of model sensitivity. These previous studies, along with results from bifurcation diagrams, regime plots and partial sensitivity analyses are informative but they are limited in scope. In particular, if one of the uninvestigated parameters in such a study is altered, the results of the study may no longer be applicable. These types of studies in which a confined set of model inputs are investigated can be interpreted as an investigation of a single virtual individual under numerous phonation regimes.

In the current paper we advocate a different sensitivity analysis and modeling paradigm in which unique instances of a selected model are used to represent a (virtual) population of subjects. Sensitivity analyses are then conducted on each member of the virtual population. This approach involves simultaneous variation of *all* model parameters and generates a “virtual population of human subjects” [10, 11] who possess a broad spectrum of possible vocal behaviors and are defined by widely spaced initial parameter sets. This approach (hereafter termed “population-based analyses”) produces an entire distribution of sensitivity values for the effect of each model input on each model output (as opposed to the traditional approach which produces a single sensitivity value for each input-output pair). This broader approach enables the identification of input parameters that have a consistent effect across the entire model’s parameter space. It also allows identification of input parameters that have erratic effects, and inputs which consistently have very minimal effect on model behavior. This type of analysis can be viewed as an experiment that has been repeatedly conducted on a representative sample of individuals so that statistical confidence is obtained, whereas traditional or partial sensitivity analyses are more akin to a single experiment conducted on a single individual.

Computational costs of population-based sensitivity analyses are high; however, they produce comprehensive understanding of global model behavior, and can therefore identify both reliable and unreliable model effects that span the entire population. In other words, results of population-based analyses can be used with confidence whereas results from partial sensitivity analysis or confined parametric variations of models (the traditional study design) do not realistically depict biological variation which exists in the real world population and are therefore limited in overall scope of relevance and applicability [12]. In particular, the traditional study design can fail to capture overall population trends [13] and extreme caution must be exercised if results from these analyses are extrapolated (i.e., generalized) to uninvestigated regions of the model’s parameter space. For additional explanation of population-based modeling and sensitivity analysis the reader is referred to [10, 11].

In this study one of the most common modeling frameworks in phonation is investigated: a two-mass model [4]. Like other lumped mass models, it makes use of a collection of discrete coupled mass-spring-damper systems subjected to an aerodynamic loading function. Numerous alterations to model geometries [14–16], aerodynamic loadings [17–19], acoustic loadings [6, 20], and contact forces [7, 21, 22] have been presented for lumped mass models and they have been used to study voice quality and singing [5, 23], voice pathologies [4, 13, 24, 25], voice instabilities [6, 26], co-articulation [27, 28], and inverse analysis from high-speed video [29, 30].

The two-mass model of Steinecke and Herzel (S&H model) was chosen for this study because of its simplicity and the fact that it has been extensively studied in normal and pathological phonation [13, 31–34]. The low computational cost of the model enabled comparison of several different sensitivity analyses, a feat that would be infeasible with more complex and computationally expensive phonation models. Furthermore, although the S&H model has been utilized in numerous studies, definitive ranges for all 16 input parameters of the model have yet to be determined.

Specific goals of this study were to (a) determine the feasibility of various sensitivity analysis approaches for voice models, (b) to determine feasible ranges for input parameters of the S&H model, and (c) to obtain a comprehensive collection of sensitivity values relating model inputs to each model output. These sensitivity values allow determination of model inputs that have consistent, significant, negligible, or erratic effects on model behavior. Furthermore, an increased understanding of input-output relationships provide a more comprehensive perspective on model behavior.

## Methods

Several types of sensitivity analyses were applied to the S&H model. These included three designed experiment type analyses and a population-based Monte Carlo type analysis [35]. Designed experiment approaches included the one at a time approach (OAT), Cotter’s method, and a quadratic response surface method (RSM). Comprehensive descriptions of each of these methods are available in standard textbooks on sensitivity analysis [36, 37], however, a brief description of each method is provided for the reader’s convenience. The following paragraph describes the S&H model, and subsequent sections describe the sensitivity analysis techniques.

**Fig 1:**
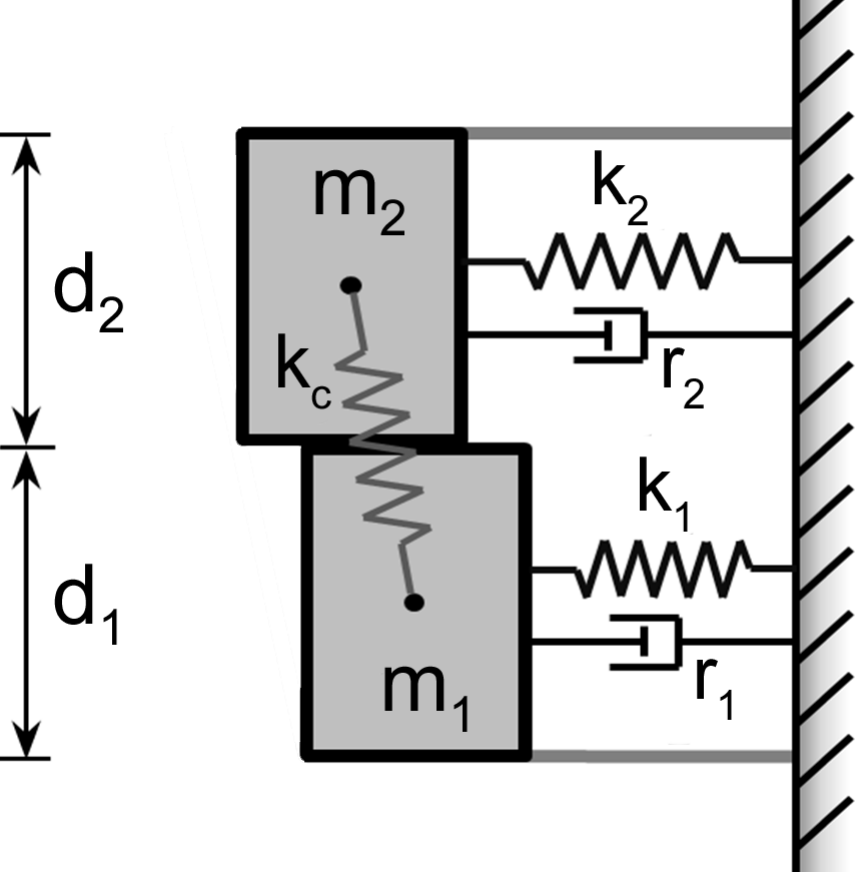
Schematic of two-mass vocal fold model of Steinecke and Herzel.

The S&H model is completely defined by 16 input parameters (Table 1 and Fig 1). The model produces a time history of glottal air-flow from which standard measures of phonation can be extracted, including fundamental frequency, mean flow rate, unsteady flow (AC flow), steady flow, maximum declination rate (MFDR), harmonic richness factor (HRF), and the difference between the first and second harmonic (H1-H2). These measures of vocal function have been used to study soft, normal, and loud voice [38], as well as pathological cases [39]. For a complete description of each of these measures see [5, 39, 40]. The current study was limited to the investigation of normal, steady phonation, as defined by the ranges for model outputs listed in Table 2. Model instances that did not produce outputs within the limits of Table 2 were excluded from the study. It should be noted that the model was symmetric, with no Coanda effect, no time varying model parameters, and no turbulence. As a result, variations in amplitude and period were minimal for each simulation.

**Table 1:** Input parameters and nominal values.

Inputs	Abbreviations	Nominal Value(s)
		(g-cm-ms unit system)
Transglottal Pressure	P	0.008 (gm/cm^/^ms^2^)
Masses	m1, m2	0.125, 0.025 (g)
Springs	k1, k2, kc	0.08, 0.008, 0.025 (g/ms^2^)
Dampers	r1, r2	0.02 0.02 (g/ms)
Physical Dimensions	d1, d2, L	0.25, 0.05, 1.4 (cm)
Collision Constants	c1, c2	0.24, 0.024 (g/ms^2^)
Initial Displacements	a01, a02	0.05, 0.05 (cm)
Reference Position	x0	0.05 (cm)

**Table 2:** Ranges for each characteristic, within which model behavior was considered as representative of normal human phonation.

Characteristic	Range	Source
Fundamental Frequency	88–263 Hz	[55]
Mean Flow Rate	50–420 cm^3^/sec	[56]
AC Flow Rate	40–800 cm^3^/sec	[55]
Maximum Declination Rate	100–1150 liters/sec^2^	[51]
Harmonic Richness Factor	-19–2.1 dB	[40]
Difference Between First and Second Harmonic	-24–11 dB	[57]

### Population-based Monte Carlo sensitivity analysis

Monte Carlo methods [36] are often referred to as a “brute force” approach since they require a large random sample from the underlying population. However, for systems which contain numerous inputs and possess nonlinear solution spaces, they are more efficient and better describe model behavior than comprehensive designed experiment approaches [35]. For example, a full factorial designed experiment approach of a model with 16 input parameters would require at least 65,536 model simulations. In this study, we were able to accurately describe the behavior of the 16 input parameter S&H model using a population of just 1000 models (i.e., virtual human subjects). A convergence test indicated that this sample size was appropriate for the S&H model.

Monte Carlo sampling was employed to obtain input parameter values for an initial population of 1000 unique virtual subjects. Sampling was performed in a coordinate system which was normalized (i.e., adjusted so that each input parameter could be fairly compared to other parameters independent of units). Normalization was performed using the nominal parameter values reported by Steinecke and Herzel [4]. The set of nominal parameter values are displayed in Table 1.

To ensure even distribution of parameter values both above and below the nominal value when sampling at broad ranges, a multi-step sampling technique was used. First, a random variable, *R*, was defined to be distributed in a uniform fashion between 1 and an upper limit, *Rmax* (in symbolic terms: *R* ∼ *U*(1,*Rmax*), where ∼ means “to be distributed as”). This variable (*R*), was defined to describe the absolute normalized distance from the nominal value. Second, a discrete Bernoulli random variable, *A* ∼ *B*(-1,1) was used to determine whether to apply *R* above or below unity. Using *r* to represent a single sampled value of *R*, *a* to represent a single sampled value of *A*, and ***P***_*0*_ to represent the vector of nominal S&H parameter values, the following equation describes how each parameter *i*, of each virtual human subject, ***P***_*subject*_, was generated:

$$P_{\mathrm{subject}\;i}=\;P_{0,i}\left(r^{a}\right)$$ (1)

For example, suppose *r* = 1.25 and *a = -1*. If this *r* and *a* value were applied to the first parameter in the model, then ***P***_*subject*,*1*_ *=* ***P***_*0*,*1*_ (1.25−^1^) = ***P***_*0*,*1*_(0.8), or 80% of the value of the first parameter of the nominal model (***P***_*0*,*1*_). For each virtual subject, unique values of *r* and *a* were chosen for each parameter, thus producing 1000 unique models. This process produces (on average) an equal number of subjects having parameters above and below each nominal value while also allowing broad sampling ranges to be used. Each virtual subject was simulated individually, and results were compared to the conditions of Table 2 to determine if each subject produced normal, realistic phonation. Sampling continued until 1000 virtual subjects were obtained, all of which exhibited normal phonation (i.e., adhered to all conditions of Table 2).

Local sensitivity analyses were performed on each of the 1000 virtual subjects. This was accomplished by individually varying each of the 16 input parameters by 1% and recording the corresponding change in behavior. When varying each of the 16 input parameters, all other parameters were held constant. To allow comparison between sensitivity values of different input-output pairs (which have different units), non-dimensional sensitivity values were calculated. This was accomplished by non-dimensionalizing both the input and output by the values at the sampled point. The following equations define non-dimensional sensitivity, S^*^ for a single output *F(x)*, and a single input *x*, where *x*_0_ is the value of *x* at the sampled point.

$$F^{*}(x)=\frac{F(x)}{F(x_{0})}$$ (2)

$$x^{*}=\frac{x}{x_{0}}$$ (3)

$$S^{*}=\frac{dF^{*}(x)}{dx^{*}}$$ (4)

Sensitivity values can be interpreted as percentage change in output due to percentage change in input (e.g., a sensitivity value of 1 indicates a 1% change in output due to a 1% change in input). This approach was extended to the six model outputs and 16 input parameters of each virtual subject.

Five different parameter ranges which represent both narrow and broad parameter bounds were used in this study. Following the conventions described above, ranges were defined as follows: *Rmax* = [1.10, 1.20, 1.50, 2.00, 5.00]. A sample of 1000 virtual subjects was obtained for each of these ranges, for a total of 5000 virtual subjects over the entire Monte Carlo study (i.e., the process described in the preceding paragraphs was replicated five times, once for each parameter range).

### Designed experiment sensitivity analyses

Designed experiment approaches utilize a predetermined set of design points to calculate sensitivities. For low dimensional spaces, this approach is often more cost-effective than the Monte Carlo approach. Brief descriptions of each of the designed experiment approaches employed in the current study are listed below.

#### Response surface method (RSM)

Response surface techniques [41] are used to obtain simplified representations of the response of complex models and are often employed in optimization problems. As is done in multiple regression, the calculation of a response surface is based on a collection of data points to which a surface is “fitted”. The location of each datum point is typically specified by a “design of experiments” to improve accuracy of the resulting fit. The D-optimality criterion [42, 43] was used in the current study to specify 231 points in the design space. Simulations were run at each of these points, and the resulting data were fit to a second-order, quadratic response surface. Sensitivity values were calculated based on the slope of the fitted response surface.

#### Cotter’s method

Cotter’s method [44] is a sensitivity analysis method used to rank model input parameters based on their influence on each model output. This approach uses a two-level fractional factorial design with all parameter values set to either the high or low extremes of parameter ranges. The first simulation (case zero) is performed with all parameters set to low values and the final simulation is performed with all parameters set to high values. Intermediate simulations consist of a single parameter being set to one extreme while all other parameters are set to the opposite extreme. Implementation of Cotter’s method required a total of 34 simulations.

#### One-at-a-time variation (OAT) method

The OAT approach [45] is a very common sensitivity analysis method in which one parameter is varied parametrically while holding all other model parameters at their nominal value. It should be noted that while this method requires very few model simulations it is not capable of detecting nonlinear effects or interactions between input parameters. Implementation of the OAT method in the current study with 2 levels for each parameter required 32 model simulations.

## Results

The designed experiment sensitivity analysis approaches failed to produce reliable sensitivity values for all but the narrowest of parameter ranges. The Monte Carlo analysis produced reliable results at all parameter ranges investigated and accurately described the global behavior of the S&H model. Results from the Monte Carlo analysis are presented first with results from the designed experiment analyses and their associated shortcomings and challenges presented second.

### Population-Based Monte Carlo analysis

**Fig 2:**
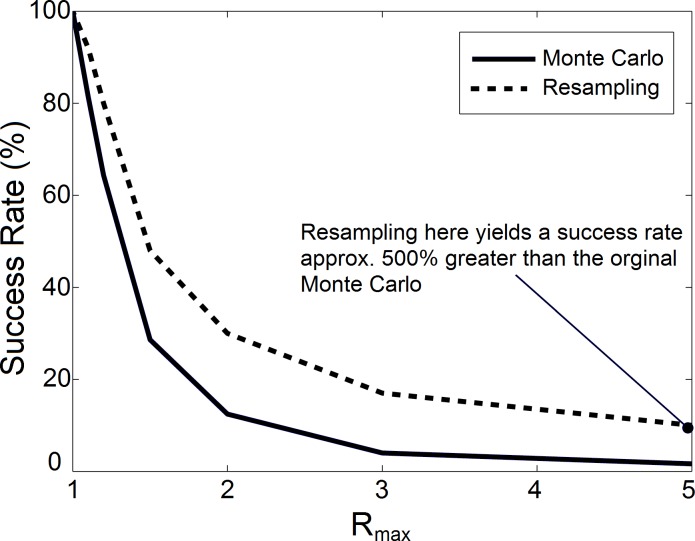
As the maximum range of input parameters was increased (increasing *Rmax*) an exponential decrease was observed in the number of model instances that produced normal realistic phonation (solid line). However, when model instances were created by sampling input parameters from a probability distribution a significant increase in the number of models producing normal, steady phonation was observed (dashed line).

The S&H model produced realistic phonation in the vicinity of the nominal input parameter set defined in [4]. As parameter ranges increased an exponential decrease in the proportion of models exhibiting normal phonation characteristics were observed (see Fig 2). For example, when *Rmax* = 5, over 60,000 virtual subjects had to be created and simulated to find 1000 virtual subjects exhibiting normal phonation.

**Fig 3:**
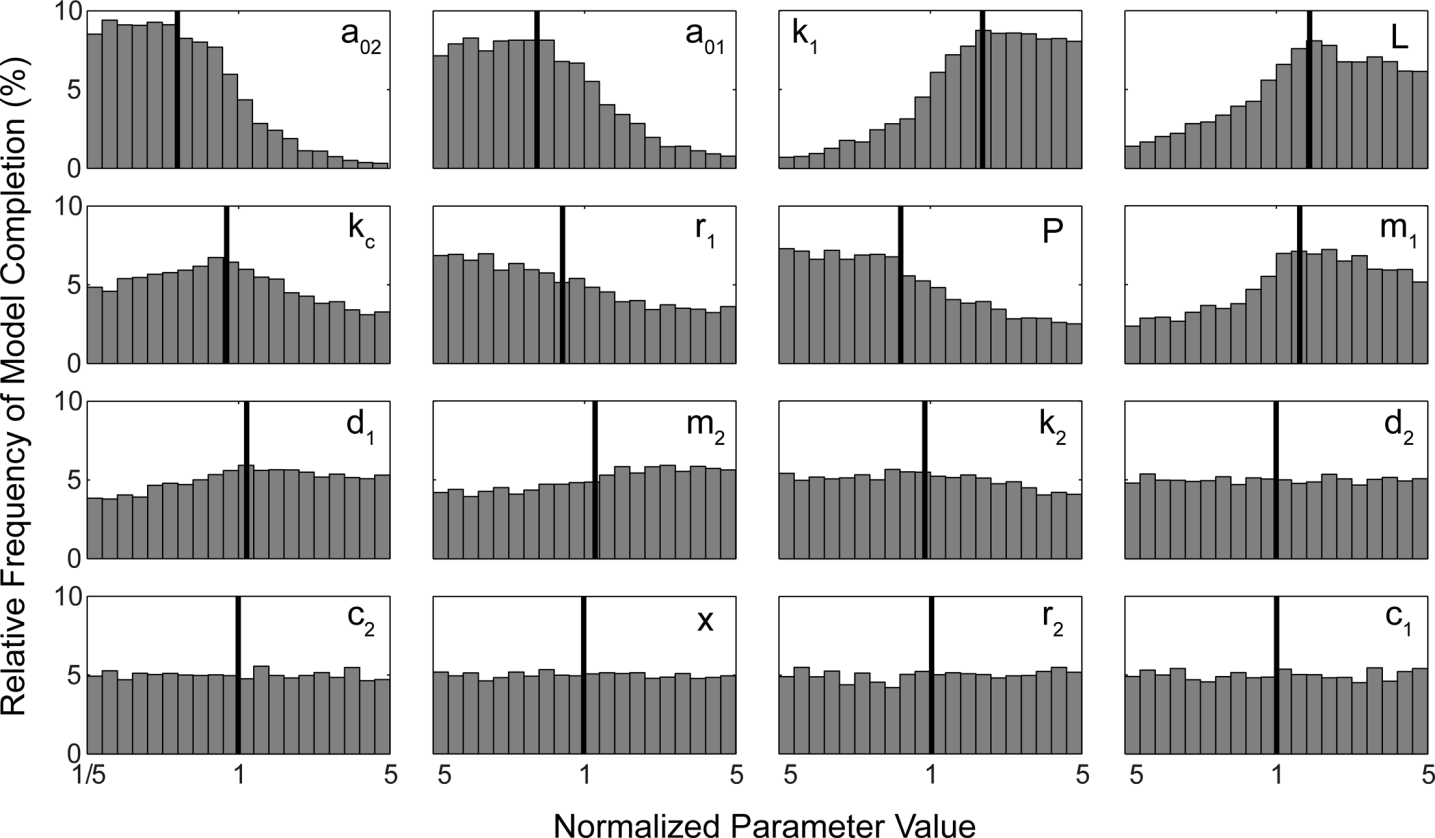
Histograms demonstrating the distribution of model success rates and their respective means (red line) are plotted for each of 16 input parameters. The range of each input parameter (horizontal axis) spans from 1/5 of the nominal value of the parameter to 5x the nominal value of the parameter. Some parameters are non-uniformly distributed (a02, a01, k1, L, Kc, r1, P, m1) indicating that certain values for these parameters increase the likelihood of nonrealistic phonation being produced. Other parameters are more uniformly distributed (c2, x0, r2, c1) suggesting that any value of these parameters is equally likely to lead to realistic phonation.

The influence of each input parameter on normal phonation was assessed by generating histograms of the distribution of normal phonation for each individual input parameter. These histograms can be seen in Fig 3. The X axis of each histogram displays the normalized parameter value while the Y axis shows the relative frequency of successful model completion. Certain parameters (namely d2, c1, c2, x0 and r2) appear as nearly uniform distributions, indicating that all parameter values within their specified ranges are equally likely to produce regular, realistic phonation. However, other parameters are clearly skewed from the original uniform distribution from which they were sampled (namely a02, a01, k1, L, kc, r1, P and m1). These parameters are more likely to produce realistic phonation when their values lie within particular regions of the specified range.

The information from Fig 3 can be used to create probability distributions to improve computational efficiency of optimization strategies which involve quasi-random sampling. For example, when sampling input parameters from a uniform distribution, with *Rmax* = 5, only 1.6% of the simulations produced normal phonation. However, the sampling success rate can be improved significantly by recursive sampling. For example, the histograms of Fig 3 were used to create probability distributions for each input parameter (via curve fitting). Points were then sampled from these new probability distributions instead of from uniform distributions. When this approach was carried out, the success rate (i.e., rate at which normal phonation was produced) increased more than six-fold from 1.6% to 10%. The dashed line of Fig 2 displays the resampling success rates at each of the five parameter ranges investigated in the current study.

**Fig 4:**
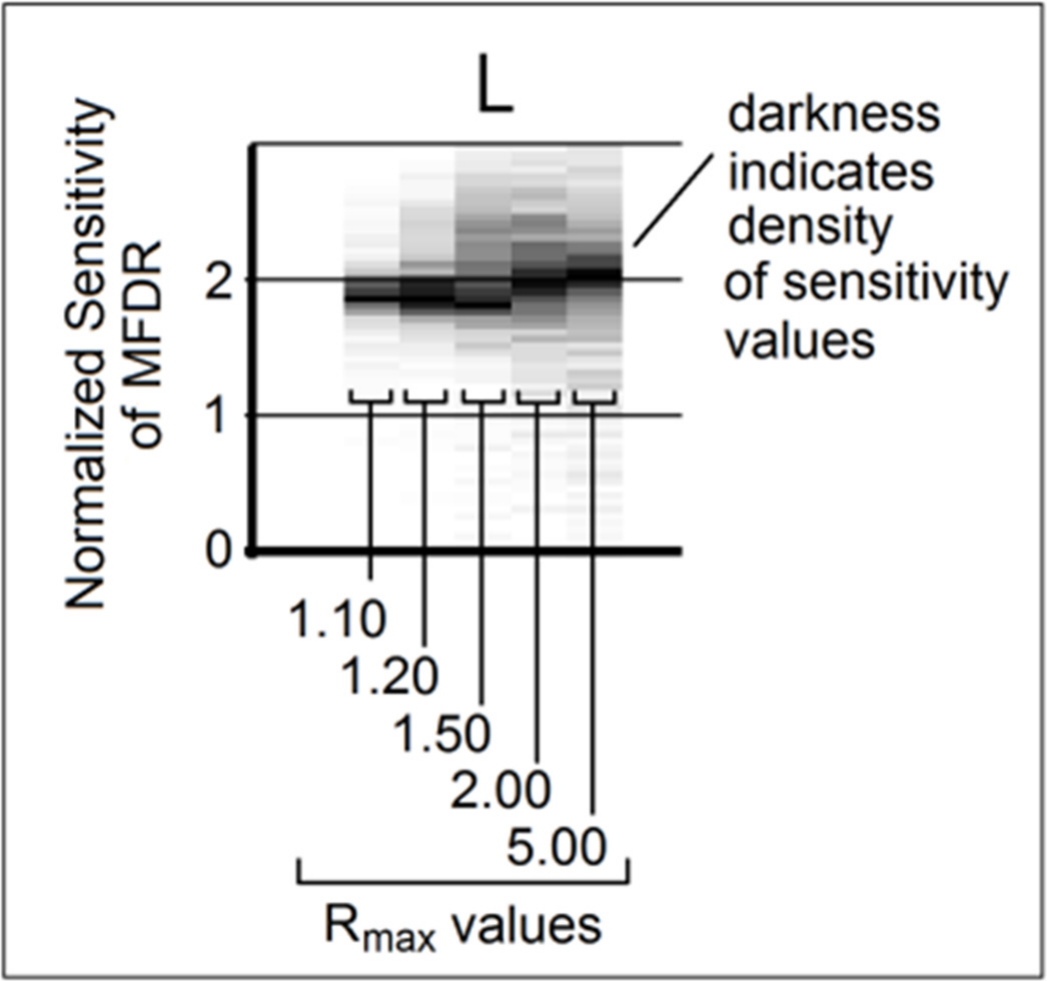
Example of a sensitivity distribution plot. The input variable is listed above the respective data (in this case, L) while the output parameter (in this case, MFDR) is listed to the left. Sensitivity data gathered from each of the parameter ranges investigated in the current study are depicted in vertical columns, as shown above. The Y axis indicates the non-dimensional sensitivity value. Grayscale intensity indicates the relative density of sensitivity values (i.e., the most common sensitivity values are the darkest).

**Fig 5:**
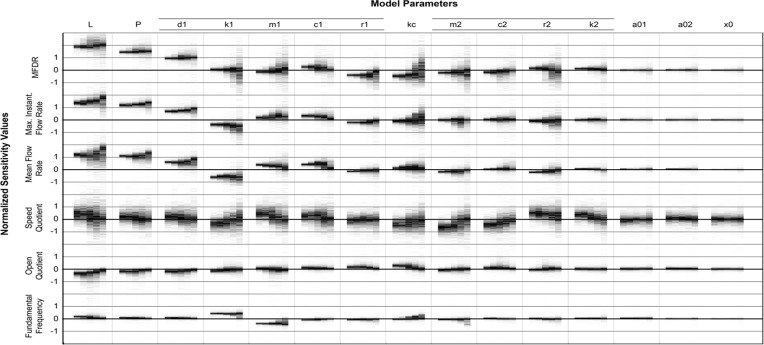
Multi-dimensional sensitivity distribution plot showing sensitivity distributions for each input-output pair of the S&H model and for various ranges of Rmax. Both Fig 4 and the text describe how to correctly interpret the sensitivity distribution plots. Along the X axis input parameters are listed from left to right in order of most influential to least influential. On the Y axis model outputs are ranked from top to bottom according to mean sensitivity.

A total of 480,000 unique sensitivity values (6 outputs × 16 inputs × 5 parameter ranges × 1000 virtual human subjects) were calculated as part of the Monte Carlo approach. A new visualization technique was developed to enable the presentation of these data: the multi-dimensional sensitivity distribution plot (MDSD plot). The MDSD is illustrated in Fig 4 for a single input-output pair. The Y axis displays the non-dimensional sensitivity value, and sensitivity histograms for each of the 5 parameter ranges are presented as vertical bands along the X axis. The intensity of each pixel (i.e., shade from white to black) in each histogram is related to the frequency at which that particular sensitivity value occurred in the virtual population of subjects (i.e., the most commonly encountered sensitivity values are the darkest regions in each column of pixels). Thus, darker regions represent areas of high relative density and the vertical position of the region represents the value of the most commonly encountered sensitivity values. Fig 5 summarizes the entire sensitivity data set (480,000 sensitivity values) across 5 critical dimensions: input parameter, model output, sensitivity value, range (Rmax value), and relative frequency. The model input parameters listed at the top of Fig 5 are ordered from the most influential (left) to the least influential (right). Model outputs listed on the left of Fig 5 are ranked from top to bottom according to mean sensitivity.

The MDSD plot (Fig 5) allows one to quickly gain meaningful insights about the behavior of the S&H model. For example, input-output pairs that have a consistent effect across the entire sampled population can be identified by histograms with dark and narrow bands. Input-output pairs that have relatively little effect on the model are characterized by narrow histograms centered on 0. The Fig also demonstrates that variance in sensitivity values between subjects depends greatly upon the output type: nearly all subjects demonstrated consistent sensitivity values with regards to fundamental frequency while sensitivity values related to speed quotient vary significantly across the population. Interestingly many input-output pairs exhibit sensitivity values both above and below zero. In other words, certain subjects demonstrate a positive relationship between a given input and output while other subjects obtained from the same population demonstrate a negative correlation between the same given input and output. This was an unexpected result that demonstrates the complexity of model dynamics, the semi-chaotic behavior of phonation models, and that large errors can be produced by generalizing model behaviors based on limited sampling (as done in partial sensitivity analyses). Sensitivity distributions that include both positive and negative sensitivities are indicators of strong nonlinear interactions within the model that are prevalent across broad ranges of input parameters. In general, the variance of sensitivity values increased with parameter sampling range.

Fig 5 also demonstrates that the average value of parameter sensitivities vary based on output type and that many input-output relationships agree with prior research. For example, fundamental frequency is most sensitive to m1 and k1 (which are known to relate to fundamental frequency) while both maximum instantaneous and mean flow are most sensitive to vocal fold length (L) and subglottal pressure (P), as predicted by the Bernoulli flow model. As with L and P, larger areas on the medial surface (d1, x0, L) result in higher flow and MFDR. The width of the inferior mass (d1) determines the surface that controls the applied fluid loading, thus being proportional to the energy transfer and in turn creating larger amplitude of oscillation. On the other hand, the stiffness (k1) of the same mass is inversely related to the amplitude of displacement, thus producing the opposite effect of d1. Vocal fold length (L) and transglottal pressure (P) were found to be the most influential parameters overall followed by the parameters associated with mass one (d1, k1, m1, c1, r1), followed by the parameter that links mass 1 to mass 2 (kc), followed by the parameters related to mass 2 (m2, r2, c2, k2).

The MDSD plot of Fig 5 also reveals that certain parameters have a minimal effect on all model outputs (see for example a01, a02, and x0). It is interesting to note that although a01 and a02 have minimal effect on actual phonation outputs, these two parameters do significantly affect the success rate of the model (i.e., contribute to the likelihood of the model producing normal phonation) (see Fig 3).

**Fig 6:**
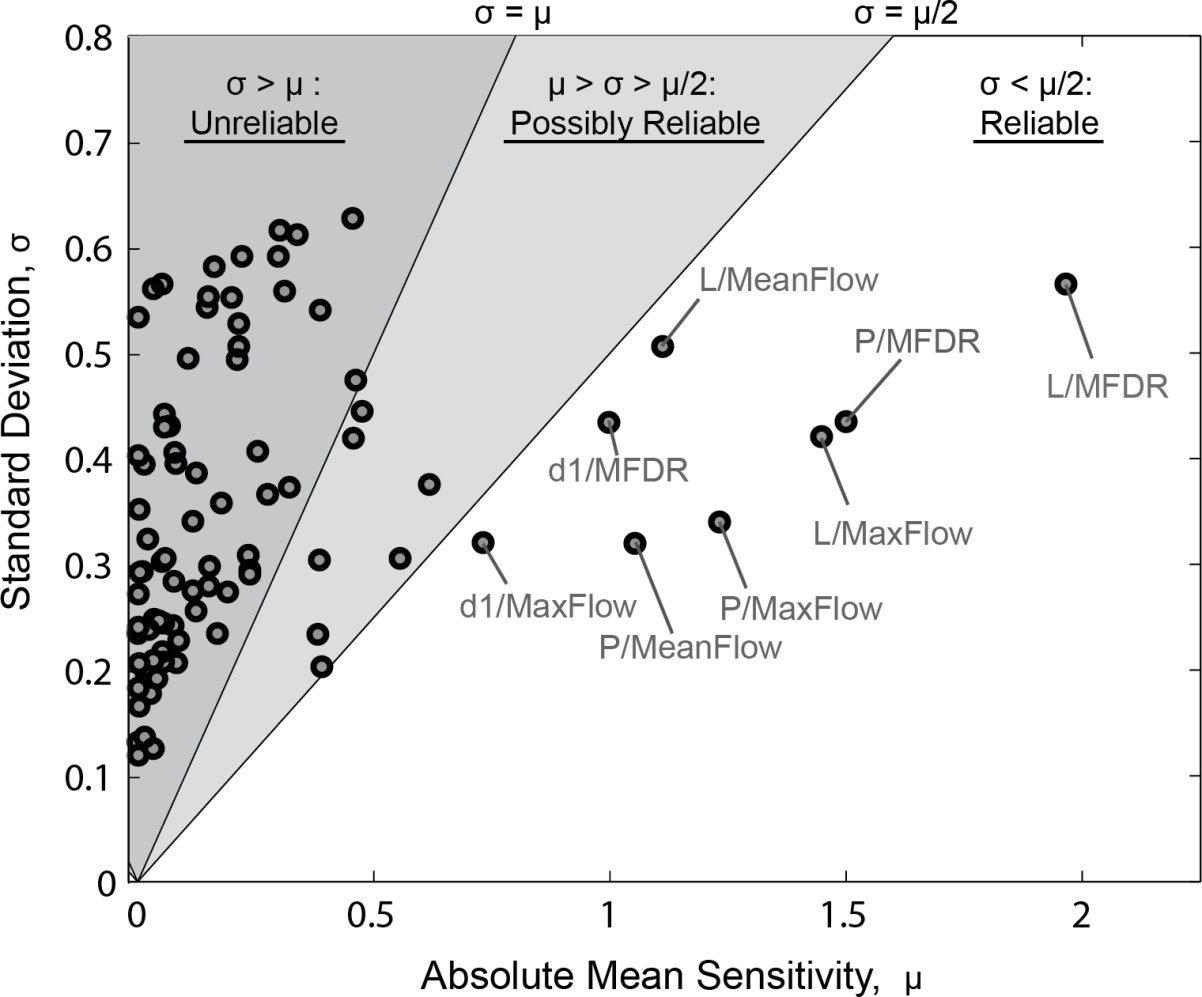
Plot of mean versus standard deviation of sensitivity values for each input-output pair (*Rmax* = 1.5). The shaded regions represent sensitivity values in which the mean is less than 1 (dark grey) or 2 (light grey) standard deviations and thus are highly variable. The vast majority of sensitivity values lie within these regions. Consistent sensitivity values are labeled in the unshaded region.

While the MDSD effectively communicates a large amount of information, and visually describes the model behavior across a population, no single figure can adequately describe a 16-dimensional space. For example, Fig 5 does not display any type of statistical measure and it is therefore not immediately obvious which model inputs have statistically significant or reliable model effects. To address this shortcoming, the mean and standard deviation of sensitivity values for each input-output pair were computed and plotted. Fig 6 shows the mean vs. standard deviation plot for *Rmax* = 1.5. The shaded regions of this figure represent areas in which the ratio of standard deviation to the absolute mean sensitivity (i.e., the coefficient of variation) is greater than 0.5. In general, points within the shaded regions represent sensitivities that are not consistently above or below 0 (i.e., not reliably above or below zero). On the other hand, input-output pairs with a high mean sensitivity and low standard deviation represent significant effects that are consistent across the entire population. These pairs are plotted in the white region of Fig 6. The effects of these pairs are consistent across virtually all subjects. On the other hand, input-output pairs with high standard deviations and relatively low means represent unpredictable sensitivities or behaviors which vary from person to person (i.e., effect cannot be generalized to the population).

Presenting data in this manner allows one to quickly determine which input-output pairs are consistently related across the entire population. Perhaps the most striking feature of Fig 6 is that the vast majority (91%) of all input-output pairs lie within the shaded regions, thus demonstrating the highly variable and nonlinear nature of the S&H model’s solution space (i.e., the majority of input parameters have unpredictable effects on model outputs). Only 8 input-output pairs (8%) have a consistent effect across the sampled population. This demonstrates the danger of conducting traditional sensitivity analyses or partial parameter investigations of phonation model’s and generalizing their results to unexplored regions of the models parameter space. Such generalizations could be wrong 91% of the time due to strong interactions between model parameters and the semi-chaotic nature of phonation.

Tables 3 and 4 summarize key findings observed in Figs 5 and 6 by listing specific input-output pairs that have a reliably significant or reliably insignificant effect on the model. The median sensitivity values as well as the 5^th^ and 95^th^ percentile sensitivity values (averaged across all parameter ranges) are listed for each input-output pair. Medians and percentiles are reported instead of averages since non-normal distributions were often encountered.

**Table 3:** Most influential input-output pair sensitivities.

Input		Output	5%-ile	Median	95%-ile
L	→	MFDR	1.25	1.94	2.77
P	→	MFDR	1.00	1.48	2.08
d1	→	MFDR	0.47	0.98	1.58
L	→	Max Flow	0.93	1.42	2.03
P	→	Max Flow	0.89	1.22	1.64
d1	→	Max Flow	0.38	0.71	1.12
k1	→	Max Flow	-1.06	-0.43	-0.01
L	→	Mean Flow	0.29	1.15	1.80
P	→	Mean Flow	0.60	1.07	1.44
k1	→	Mean Flow	-1.10	-0.61	-0.18
d1	→	Mean Flow	0.11	0.57	0.94
c1	→	Mean Flow	0.00	0.40	0.74
k1	→	Frequency	0.12	0.40	0.59
m1	→	Frequency	-0.59	-0.39	-0.08
L	→	OQ	-1.22	-0.36	-0.01

**Table 4:** Least influential input-output pair sensitivities.

Input		Output	5%-ile	Median	95%-ile
x0	→	Frequency	-0.06	0.00	0.06
a02	→	Frequency	-0.07	0.01	0.08
a01	→	Frequency	-0.05	0.04	0.15
c2	→	Frequency	-0.18	0.01	0.14
k2	→	Frequency	-0.13	0.03	0.16
x0	→	Mean Flow	-0.10	0.00	0.09
a02	→	Mean Flow	-0.12	0.03	0.19
x0	→	OQ	-0.16	0.00	0.15
a01	→	OQ	-0.16	0.01	0.19
a02	→	OQ	-0.13	0.03	0.20
x0	→	Max Flow	-0.17	0.00	0.15
x0	→	MFDR	-0.19	0.00	0.20

### Designed experiment sensitivity analyses

The designed experiment sensitivity analyses only produced acceptable results in very narrow regions of the model’s parameter space located near the nominal parameter set. When investigating broad parameter ranges model simulations would frequently fail to produce normal phonation thus preventing computation of sensitivity values (i.e designed experiment approaches rely upon obtaining normal phonation at *all* of the pre-determined sample points).

The response surface method proved to be problematic at all of the parameter ranges investigated except for *Rmax* = 1.10. The accuracy or goodness of fit for the response function within this range was tested by comparing it to additional simulations of the S&H model at 100 randomly selected points. The average maximum error was less than 5% suggesting that the response surface was indeed a good fit.

Cotter’s method and OAT failed at each of the five parameter ranges investigated in the current study. The maximum parameter range for which Cotter’s method was able to calculate sensitivity values was ±2% of the nominal parameter value. The maximum parameter range for OAT was ±7% of the nominal parameter value. Both methods produced results which were generally in agreement with each other as well as with the Monte Carlo results. However, the narrow regions in which these methods could be successfully applied were unsuitable for the purpose of this study, which was to investigate trends across a broad population. Results from these methods are therefore not presented in detail.

Table 5 lists the advantages and disadvantages of each sensitivity analysis method investigated. The most surprising feature of Table 5 may be the extremely small percentages of the design space which were analyzed by the designed experiment methods. For example, ±7% of the nominal parameter values may at first seem adequate, but such a domain represents much less than 1 trillionth of 1 percent of the domain analyzed by the Monte Carlo method when *Rmax* = 5. This result can be verified by calculating the volume of the model’s parameter space interrogated using each parameter range.

**Table 5:** Comparison of sensitivity analyses.

	Required		% Design	
	Model Evaluations	Reliability	Space	Interactions
			Analyzed	
**Cotter's**	n+1 = 17	Low	1e-32	Yes
**OAT**	2n+2 = 34	Low	1e-23	No
**RSM**	Variable. For the current study, n = 231	Dependent on fit	1e-21	Yes
**Monte Carlo**	Variable, for the current study n = 17,000 for each range, for a total of 85,000.	High	100	Yes

n = number of input parameters

## Discussion

### Ranges for input parameters of the S&H model

One purpose of the current study was to determine feasible ranges for input parameters of S&H model. Unfortunately, a concrete or discrete input parameter boundary beyond which normal phonation is not produced was not found for the S&H model. Even in the broadest parameter range explored in this study, none of the parameters exhibited a region in which normal phonation was not achieved. As the distance of any given parameter's value deviated from the nominal value, the probability of the model producing normal phonation was reduced but the probability never reached 0. In other words, an incredibly large range of broadly spaced initial parameter sets can lead to normal phonation. This type of behavior is typical of many biological systems and such systems are better explained by probabilities and distributions than by discrete values or averages [46]. Therefore, feasible ranges for input parameters to the S&H model are presented as distributions and not as set values beyond which normal phonation is not encountered. These distributions are presented in Fig 3. In an ideal situation the distributions of input parameters would be based on empirically derived probability distributions (as opposed to model derived probability distributions). However, this study was performed precisely because such data are not available for the S&H model. Observation of Fig 3 reveals that the nominal set of S&H model parameters is *not* the set of parameters most likely to lead to normal phonation. Rather, the statistical mode of each histogram in Fig 3 represents the parameter set most likely to lead to normal phonation.

The parameter distributions of Fig 3 were useful in reducing the computational burden of subsequent sampling trials. For example, when sampling from a uniform distribution with *Rmax* = 5, the success rate (percent of virtual subjects that produced normal phonation) was 1.6%. However, when the probability distributions of Fig 3 were used as a sampling basis, the success rate increased by more than 6 fold to an overall rate of 10% (Fig 2). Future modeling studies can be improved by using cumulative distribution functions to select sampling points instead of sampling from a normal or uniform distribution. This approach can reduce the number of simulations required to achieve a desired population of virtual subjects and when performing optimizations which rely on selective sampling.

### Sensitivity values for the S&H model

A second purpose of this study was to determine sensitivity values for the S&H model. The holistic sensitivity analysis approach undertaken in the current study provides a more comprehensive description of the S&H model’s solution space than provided by studies employing a standard modeling paradigm (i.e., using partial sensitivity analyses or regime plots to investigate different phonatory regimes of a single virtual subject). For example, prior studies [13, 31–33], have observed that isolated regions of the S&H model’s solution space exhibit complex behaviors and nonlinear interactions between input parameters. The population-based analysis presented in this study demonstrates that these nonlinear behaviors dominate the entire solution space of the S&H model in such a way that the vast majority of model inputs have unpredictable effects on model outputs (see Fig 6). Population-based analyses avoid serious errors associated with extrapolating results into unexplored regions of a model’s parameter space and present sensitivity values in a more correct context (i.e., as distributions and probabilities as opposed to discrete values).

This study made an assumption of sampling independence between model parameters. However, physical vocal fold parameters are known to be interconnected (e.g., changes in L affect d1, k1, m1) through physiological rules for muscle activation [47, 48]. Inclusion of physiological correlations in this study would have better represented the underlying physiology, but would have likewise prevented quantification of independent parameter sensitivities. Furthermore, sensitivity results would have been influenced by the assumed physiological relationships. With this clarification in mind, analysis of the independent parameter sensitivities computed in this study reveal several interesting insights.

We observed that all of the independent parameter sensitivities of the S&H model do not agree with prior experimental studies which have measured human phonation characteristics. For example, experimental studies have shown that female subjects have shorter vocal folds [49, 50], and therefore lower aerodynamic measures (MFDR, peak flow, mean flow, etc.), and higher fundamental frequency than male subjects [51]. The independent parameter sensitivities of the S&H model likewise indicate that changes in vocal fold length affect the aerodynamic measures and fundamental frequency. However, the degree to which fundamental frequency is influenced by vocal fold length was less significant in this study than in experimental studies involving humans. In addition, human speech typically exhibits more substantial fluctuations in frequency due to vocal fold posturing (d1, k1, m1) [52, 53] than those indicated by the independent parameter sensitivities of the S&H model. Lastly, the S&H model indicates that fundamental frequency increases with lung pressure at a rate of approximately 1 Hz per cm H2O. This is somewhat lower than has been reported with respect to human data (3–5 Hz per cm H2O) [53]. This particular discrepancy is likely due to the fact that the S&H model does not capture vocal fold elongation that is observed in humans at high transglottal pressures [53]. The other discrepancies are likely tied to the assumption of sampling independence between model input parameters. As mentioned previously, in the human condition physiological changes in vocal fold length are accompanied by other simultaneous changes to the vocal folds. The principal advantage of an independently sampled sensitivity approach is that it decouples coordinated efforts of multiple control parameters observed in humans so that the influence of each individual parameter can be determined. This is generally not possible in human studies. In the future physiological relationships (e.g., Titze and Story, 2002) could be incorporated into the sampling scheme of a sensitivity analysis of the S&H model. However, as mentioned previously, it must be realized that these physiological relationships will directly affect the sensitivity outcomes, and separating the effects of the rules that control imposed physiological relationships from the natural intrinsic model complexity becomes challenging. The independently sampled sensitivity approach presented in this manuscript is the first step toward a subsequent examination that includes parameter interdependence.

### Applying sensitivity analyses to other models

What lessons and experience in this study can be applied to more complex models of the human vocal apparatus? First, we observed that the global behavior of the S&H model could not be fully described by OAT, Cotter’s or response surface methods. This is partially due to the highly variable, multidimensional, nonlinear, bifurcated solution space of the model [4, 7], and partially due to the fact that this study restricted analysis to “normal” phonation. Designed experiment approaches provide the advantage of reduced sample sizes, but are problematic when parameter spaces are defined by numerous regimes of different behavior and when one or more tests are used to include or exclude results from the study (e.g., excluding irregular phonation). We therefore anticipate that designed experiment type sensitivity analyses of more complex voice models will likewise fail if broad input parameter ranges are investigated. It should be noted that this study originally began without a requirement of “normal” phonation. However, many parameter combinations lead to entirely unrealistic phonation or fail to produce phonation all together.

The population-based sensitivity analysis approach provided exceptionally detailed and comprehensive data, but required a total of 85,000 simulations. Such high numbers of simulation cases may not be possible for some models due to computational expense. Consequently, the majority of prior studies have conducted partial sensitivity analyses in which only a limited number of input parameters are varied over fairly small ranges. While beneficial, such results can be misleading as they describe an extremely narrow region of a highly variable, semi-chaotic design space. Such results cannot be accurately extrapolated, extended or generalized to other areas in the model’s parameter space or to the human population.

### Tradeoffs between higher order and lower order models

Complex higher order models are most suitable for understanding physical mechanisms, but are not currently appropriate for obtaining generalizable results that account for variations that occur within a population. This is primarily due to their computational expense. Frequent interactions between advanced models, reduced-order models, and population-based studies are needed to effectively translate physical insights obtained from advanced models to results that are generalizable to the human population. For example, reduced-order models can be refined to more accurately account for details revealed by more advanced models. These improved, reduced-order models can then be used to verify if the advanced physical insights provided by the more complex models persist across populations. However, the trend in recent years seems to favor the creation of more and more advanced models, with less emphasis placed on reduced-order models and sensitivity analysis studies focused on understanding model behavior. More reduction of order and sensitivity analysis studies are needed to fully utilize the insights provided by advanced models. In addition, more partial sensitivity analyses of higher order models are needed, as they provide valuable information about local model behavior.

Finally, the advantage of the population-based modeling and sensitivity analysis approach is highlighted by examining the difference between a partial multi-parameter sensitivity analysis in which a few input parameters are investigated and a comprehensive population-based multi-parameter sensitivity study in which all input parameters are varied. In a previous partial sensitivity study of a lumped mass model [8], 7 of 21 input parameters were varied while the remaining 14 parameters were held constant. The results of such a study are informative, but must be interpreted correctly. What effects might be observed if the 14 fixed parameters of [8] are varied? Results from this study suggest that a different set of 14 fixed parameters may give very different results. Unfortunately, the degree of discrepancy is unknowable unless the fixed parameters are varied. For well-behaved systems, results can reasonably be extrapolated into unexplored regions of the parameter space with minimal loss in accuracy. However, for highly nonlinear systems which can at times demonstrate chaotic behavior (such as phonation models) serious problems result from extrapolating results into unexplored regions of the parameter space. Thus, while partial sensitivity analysis models are a step in the right direction, their results cannot be used with confidence or generalized to the human population unless simultaneous variation of all parameters is considered.

## Summary and Conclusion

A comprehensive population-based sensitivity analysis of the S&H vocal fold model was conducted to provide a global description of model behavior representing normal phonation. Results demonstrate that lumped mass vocal fold models are relatively simple in their construction and simulation but they capture complex global behaviors characteristic of human phonation. These behaviors challenge normal intuition, and cannot be accurately described using single discrete values or even average sensitivity values. Consequently, results from the current study were presented in terms of probabilities and distributions which provide a more accurate and comprehensive description of model behavior. Future studies may benefit from similar population-based modeling approaches. The parameter rankings and parameter distributions reported in this study can be applied to improve the sampling efficiency of future studies which rely upon optimization of two-mass model parameters to match voice recordings.

Of the four sensitivity methods investigated (OAT, Cotter’s Method, Response Surface Methology, and population based-method), only the population-based Monte Carlo approach was able to accurately and comprehensively describe the behavior of the two mass S&H model. This approach successfully identified important input-output pairs, and revealed that the two mass model is dominated by numerous nonlinear interactions between parameters, leading to high levels of variance in the majority of outputs. It also demonstrated that very broad ranges for all input parameters of the S&H model can produce normal phonation.

Higher order vocal fold models include more physical effects and input parameters and therefore more accurately represent real world conditions. However, their computational burden prevents a comprehensive analysis of global model behaviors thus making it difficult if not impossible to generalize model results to the real world population. On the contrary, lower order models typically exhibit fewer degrees of freedom, fewer physical mechanisms and relationships, and a relatively small number of input parameters. These simplifications drastically reduce computational expense. Comprehensive population-based sensitivity analyses of these models allow model results to be more readily generalized across the real world human population. In the future, frequent interactions between advanced models, reduced-order models, and population-based studies can provide an effective path for translating the physical insights obtained in advanced models to results that are generalizable across a population. Three-dimensional lumped mass type models in particular are sufficiently complex to mimic clinical phenomena, while remaining computationally efficient enough to enable their solution spaces to be quantified via sensitivity analyses. Such models, continuously informed and improved by more complex models, may be prime candidates for future studies seeking to contribute to advances in voice and speech therapy.

Douglas Osheroff, the Nobel Prize winning physicist has said, “we can never understand our scientific tools too well” [54]. As the field of voice production continues to generate complex biomechanical models, we must continuously reflect on our capability to comprehensively analyze, predict, and quantify the behavior of our models.
