# Correction: Comprehensive, Population-Based Sensitivity Analysis of a Two-Mass Vocal Fold Model

**DOI:** 10.1371/journal.pone.0156569

**Published:** 2016-05-27

**Authors:** Daniel Robertson, Matías Zañartu, Douglas Cook

There are errors in the Funding section. The correct funding information is as follows: This research was supported by funding from New York University Abu Dhabi and Comisión Nacional de Investigación Científica y Tecnológica (Republic of Chile), grants FONDECYT 1151077 and BASAL FB0008.
